# Chlorhydrate of Pereirine in Malarial Fevers

**Published:** 1886-06

**Authors:** Clemente Ferreira


### Chlorhydrate of Pereirine in Malarial Fevers.

Pereirine is an alkaloid derived from the bark of a shrub belonging to the Apocynaceæ family, the *Geissospermum laeve*, a native of Brazil. The bark of this plant has a very bitter taste, and is frequently used in the treatment of malarial fevers, the patient being usually bathed in a decoction of the bark.

The late Brazilian chemist, E. Correia, was the first to extract from the bark of the *Pau-pereira*, as it is commonly called in its native country, an active substance of an alkaloidal nature, which he called *Pereirine*. This substance has since been used by many Brazilian practitioners in a variety of affections with excellent results. Dr. Domingos Freire has succeeded in obtaining a combination of pereirine with the acids, and its use has been thereby greatly extended. The muriate and valerianate of pereirine are soluble, and chemically pure, in this respect being much superior to the alkaloid. I much prefer the muriate of pereirine, as it is very soluble in water, and have used it in many cases, always with marked success. Patients whose fever resisted the salts of quinine were promptly cured by the use of pereirine.

I have recently obtained a brilliant victory by the use of this salt. My patient was a woman, who for three months had suffered from a severe attack of malarial fever of an intermittent type. All the usual therapeutic agents have been employed without success. A change of climate had been tried without avail, the patient continuing to have the intermittent attacks.

On examination, I found a marked enlargement of the spleen and liver, with jaundice and great emaciation. I prescribed chlorhydrate of pereirine in doses of two grammes (gr. 30), to be divided in four capsules, and taken four hours before the usual time for the beginning of the attack. This medicine was preceded by a dose of calomel.

As a result the patient had no attack on that day. But the following day the fever returned, no pereirine having been administered. On the following day another dose was given as before, and on a few subsequent days gramme doses were given.

The malarial fever has not since appeared, and the patient is well. In many other cases I have found this drug valuable, especially in the intermittent fevers, and the malarial fevers of children. When the salt is to be given to children, I usually prescribe it in syrup of orange peel.

We all meet with cases of malarial poisoning in Brazil which resist quinine and arsenical treatment. In these cases the use of chlorhydrate of pereirine will achieve a brilliant success, and the practioner will loudly praise this invaluable drug, which must henceforth occupy an important place in the therapeutics of malaria.—*Dr. Clemente Ferreira, in Bull. Gen. de Therapeutique.*

